# Cost effectiveness of community-based and in-patient therapeutic feeding programs to treat severe acute malnutrition in Ethiopia

**DOI:** 10.1186/1478-7547-10-4

**Published:** 2012-03-19

**Authors:** Asayehegn Tekeste, Mekitie Wondafrash, Girma Azene, Kebede Deribe

**Affiliations:** 1 Jimma University Faculty of Public Health, Jimma, Ethiopia; 2 Jimma University Faculty of Public Health, Department of population and family health, Jimma, Ethiopia; 3 Jimma University Faculty of Public Health, Department of Health Planning and management, Jimma, Ethiopia; 4 Jimma University Faculty of Public Health, P.O.Box 2082 code 1250 Addis Ababa, Ethiopia

**Keywords:** Severe acute malnutrition, Community- based therapeutic care, Therapeutic feeding center, Cost-effectiveness

## Abstract

**Background:**

This study estimated the cost effectiveness of community-based therapeutic care (CTC) for children with severe acute malnutrition (SAM) in Sidama Zone, Ethiopia compared to facility based therapeutic feeding center (TFC).

**Methods:**

A cost effectiveness analysis comparing costs and outcomes of two treatment programmes was conducted. The societal perspective, which considers costs to all sectors of the society, was employed. Outcomes and health service costs of CTC and TFC were obtained from Save the Children USA (SC/USA) CTC and TFC programme, government health services and UNICEF(in kind supplies) cost estimates of unit costs. Parental costs were estimated through interviewing 306 caretakers. Cost categories were compared and a single cost effectiveness ratio of costs to treat a child with SAM in each program (regardless of outcome) was computed and compared.

**Results:**

A total of 328 patient cards/records of children treated in the programs were reviewed; out of which 306 (157 CTC and 149 TFC) were traced back to their households to interview their caretakers. The cure rate in TFC was 95.36% compared to 94.30% in CTC. The death rate in TFC was 0% and in CTC 1.2%. The mean cost per child treated was $284.56 in TFC and $134.88 in CTC. The institutional cost per child treated was $262.62 in TFC and $128.58 in CTC. Out of these institutional costs in TFC 46.6% was personnel cost. In contrast, majority (43.2%) of the institutional costs in CTC went to ready to use therapeutic food (RUTF). The opportunity cost per caretaker in the TFC was $21.01 whereas it was $5.87 in CTC. The result of this study shows that community based CTC was two times more cost effective than TFC.

**Conclusion:**

CTC was found to be relatively more cost effective than TFC in this setting. This indicates that CTC is a viable approach on just economic grounds in addition to other benefits such improved access, sustainability and appropriateness documented elsewhere. If costs of RUTF can be reduced such as through local production the CTC costs per child can be further reduced as RUTF constitutes the highest cost in these study settings.

## Introduction

Severe acute malnutrition (SAM) is defined by a very low weight for height (below -3 z scores of the median WHO growth standards), by visible severe wasting, or by the presence of nutritional oedema. In children aged 6-59 months, an arm circumference less than 115 mm is also indicative of severe acute malnutrition. SAM affects approximately 20 million children under five years of age and contributes to more than 1 million child deaths in the world each year. Global moves against the high prevalence rate of malnutrition have shown remarkable progress, although there are still some disturbing exceptions in many developing countries particularly in the Sub-Saharan Africa [1-3]. Malnutrition, severe or otherwise, is estimated to be a contributing factor in over 50% of child deaths [4], and it is estimated that the reduction in child mortality and morbidity (i.e., loss of disability-adjusted life-years [DALYs] averted) if malnutrition were eliminated would be at least one-third [5,6].

Therapeutic Feeding in emergencies relies on traditional inpatient-Therapeutic Feeding Centers (TFCs) as the primary mode of interventions. TFCs provide intensive, high quality care for severely malnourished individuals [7]. TFCs are large, in-patient centers where SAM patients are admitted for a period of 21 days or longer. TFCs make use of the WHO guideline for treating acute severe malnutrition [8]. Mothers or caregivers are often required to stay with their malnourished children for three weeks or longer in the TFC [9]. The limitations of a hospital-based approach for a condition affecting large numbers of children, particularly when hospital capacity is poor, have been recognized for more than 30 years [10,11]. Moreover, hospital stays of several weeks for a child and mother are disruptive for families, especially when the mother has other children at home or when her labor is essential for the economic survival of the household. As a result, hospital-based management of severe malnutrition was perceived as efficacious, but not effective, on a large scale, either as part of routine health services or in emergencies [12].

The traditional inpatient Therapeutic feeding centers are which were intended to rehabilitate children with severe malnutrition. These children were identified by a weight-for-height ratio less than 70% of the median for a reference population, bilateral pitting edema (severe fluid retention in the feet and extremities), or a mid-upper-arm circumference (MUAC) less than 110 cm. In a therapeutic feeding center, the World Health Organization (WHO) protocol for the management of severe malnutrition is to provide a basic medical package to treat infection and to use therapeutic milk based formulas for nutritional rehabilitation. These milk-based formulas, F75 and F100, were used for severely malnourished children.

In 2005 a new model of delivering care has been proposed, called community-based therapeutic care (CTC) that is designed to address the limitations of inpatient care [13]. CTC programs use decentralized networks of outpatient treatment sites (usually located at existing primary health-care facilities), small inpatient units (usually located in existing local hospital facilities), and large numbers of community-based volunteers to provide case detection and some follow-up of patients in their home environments. Patients with severe malnutrition, with good appetite, and without medical complications are treated in an outpatient therapeutic program (OTP) that provides ready-to-use therapeutic food (RUTF) and medicines to treat simple medical conditions. The food and medicines are taken at home, and the patient attends an OTP site weekly or fortnightly for monitoring and resupply. Severely malnourished persons with medical complications and/or anorexia are treated in an inpatient stabilization center (SC) where they receive standard World Health Organization (WHO)-recommended initial care until they have enough appetite and are well enough to continue with outpatient care [14].

In 2007 multilateral UN agencies endorsed the approach. They acknowledge that treatment has been restricted to facility-based approaches, greatly limiting its coverage and impact. They endorsed community-based management of acute malnutrition, stating that large numbers of children with severe acute malnutrition can be treated in their communities without being admitted to a health facility or a therapeutic feeding centre [15]. In recent years Community-based management of acute malnutrition (CMAM) is widely used term which evolved from CTC and suites both emergency and development contexts. The approach focuses on integration of management of acute malnutrition into the existing health system [16].

Ethiopia has been experiencing drought and chronic food insecurity for the last four decades. The situation in the last few years has been serious. In 2002/2003 droughts affected areas faced particularly high acute malnutrition rates ranging from 10 to 34% GAM (global acute malnutrition) and 1 to 8% SAM (severe acute malnutrition). Some 460,000 children or 15% of the total under the age of five years were estimated to have suffered from acute malnutrition and 60,000 (2%) from severe acute malnutrition [17].

One of the emergency support programs that have been put in place was the establishment of inpatient-Therapeutic Feeding Centers (TFCs) by various NGOs in various parts of the country. In 2003, over 40 TFCs opened in the country, 26 were located in SNNPR [18]. Save the Children USA's Emergency Health and Nutrition Program (EHNP) was actively involved in life saving emergency activities during the 2002/2003's food crisis in the country, particularly in SNNPR state. It had opened many TFCs for assisting drought victims [19]. However, after about five months of operation high number of case loads, the need to reach more cases with otherwise limited capacity of the centers to care for more children and the potential for disease outbreaks in the centers and the Regional Health Bureau's concerns on the increasing number of TFC prompted SC/USA to pilot CTC in three woredas of SNNPR [20,21].

Operational level cost data were scarce and there were very little progress in this dimension to date. Some experts may view CTC as an expensive endeavor. However, evidences from the operational level suggest the opposite [22-24]. A report by Khara & Collins [24] stated that comparing costs of TFCs and CTC programs is difficult for several reasons. They suggested, "in order to satisfactorily provide a cost comparison, a comprehensive piece of work needs to be undertaken, taking into consideration both quantitative and non quantitative factors that influence direct and indirect costs to programs and their beneficiaries" [24]. A comprehensive cost-effectiveness study by Ashworth & Khanum [25] compared the costs for three delivery systems: inpatient care, day care and domiciliary rehabilitation care. The study showed that domiciliary rehabilitation was the most cost-effective [24]. Similar findings were reported in other studies [26,27].

There are only few studies on the cost effectiveness of CTC done until now. However better information on cost effectiveness of both TFC and CTC is required to guide policy decision [22,26]. This information is particularly important for the government and donors who would like to scale up their interventions and improve the transition from emergency to long term health development [23]. Therefore this study was conducted to 1) determine the average cost of treatment of a severely malnourished child in TFC and CTC in SNNPR, 2) to determine the effectiveness of TFC and CTC as measured by the clinical outcomes and 3) to determine and compare the cost effectiveness of the two programs.

## Methods

### Study design and settings

The study was a retrospective comparative cost- effectiveness evaluation of therapeutic feeding programs in emergencies. The interview and data review were conducted in Shebedino woreda of the Sidama zone in the SNNPRS from February to April 2007. The woreda is located south of the capital of the regional state-Awassa. It is one of the nineteen Woredas of Sidama Zone and it has 50 rural and 3 urban kebeles with an estimated population and population density of 315,354 and 630 people per Km2 respectively. Farming, which combines both crop cultivation and livestock rearing, are the major economic activities for 98% of households living in rural areas of the district. An average farmland shared by a household is less than or equal to 0.5 hectare.

In years (2000-2005), there have been unfavorable rainfall patterns, hampering crop production that resulted in serious food shortage in lowland and mid highland areas of the district. In 2004, the information obtained from the Woreda DPPC office revealed that out of those 50 rural kebeles, 32 kebeles were in need of emergency food aid. Of those 32 kebeles, 22 of them had been receiving general ration from the regional DPPC. SC/USA started running CTC program in the Shebedino Woreda in July 2005 [28].

Morocho TFC was opened in July 24, 2003 and closed in January 28, 2004. It was opened according to the recommendations of the findings from the rapid assessment conducted by SC/EHNP and government on 4-June-03. The TFC was found 24 kms south of Awassa and 4 kms from Leku town, the seat of the Shebedino woreda administration. The TFC was located within a health post compound. Initially the center's capacity was planned to accommodate 100 children. However, as there were 122 children admitted within the first week, the capacity was revised to accommodate 200 children [29].

The Shebedino Woreda CTC program was opened on 25th of July 2005 following the nutrition survey result conducted in May 2005, which had shown GAM of 16% with aggravating factors. The program had established one Stabilization Centre at Leku health centre and 8 CTC sites [28].

### Sample size determination

The sample size for the study was determined using the sample size determination formula for comparing two population means. The Bangladesh study showed that, the mean parental costs (wage loss) in inpatient care and domiciliary care (as a proxy to CTC) were US$ 2.6 ± 3.0 and US$1.6 ± 1.0 respectively [25]. For this calculation, the variance that gives the highest sample size i.e. 3 was taken from the above study. The least mean difference sought to be detected is one US Dollar (1$). Using 95% confidence interval and power of 80% (ß = 0.02), the sample size for each group becomes 142 making the total 284 with 1 to 1 ratio of TFP and CTC caretakers. Taking a 15% allowance for non-response, missed cases and out migration, the final sample size was determined at 328.

### Selection of the programs to be studied

Morocho TFC was selected from many others because it was the only program that had functioned long as a TFC and was run by SC/USA until its closure. The rest either made transitions from TFC to Stabilization centers for CTC programs or they were handed over to partners (government and other NGOs) shortly after start up. On the other hand the community based therapeutic program (CTC) in the same woreda which was started on 25th of July 2005 and is functioning until the date of data collection is included in the study to enable sound comparison (in terms of their location, population characteristics, prevalence of acute malnutrition) between the programs. Moreover, the inclusion of Shebedino CTC program in this study offered a valuable base for conducting analysis on a relatively recent data set.

In this analysis the cost of TFC was taken for the period of July 24, 2003 to January 28, 2004 and for CTC July 2005 to April 2007.

### Sampling procedure

Stratified random sampling was employed to select the study subjects (patient cards and caretakers). First the list of kebeles where the children served by the respective programs came from were stratified into near, medium and far to the Morocho TFC based on the information from the qualitative study. The calculated sample size was allocated proportional to the size of children treated in each stratum. For these selected study subjects patient card review was done. The caretakers of these selected children were interviewed for the opportunity costs they incurred tracing back to their households.

### Data collection instruments

The researchers have developed data collection instruments (checklists and formats). Health extension workers and CHAs who were able to speak the local language, and who were residence of the study kebeles were recruited and collected the data for the household survey of this study.

A semi-structured guide was used to collect data on the costs attributable to care takers from interviews and from Focused Group Discussions (FGD). A health professional who had previous experience in conducting FGD and who also speaks the local language facilitated the discussions in the presence of the one of the investigators. Ten people were interviewed as key informants. Two FGDs each for TFC and CTC caretakers from Telamo and Remeda kebeles were conducted.

The main purpose of the interviews and FGDs was to estimate the age at employment in the area, determine the wage rate & productivity during specific season in a day and then use it in the calculation and valuation of productivity lose or wage loss, time spent to reach the programs site, transportation cost and waiting time to get services.

### Administrative data and patient cards

Administrative records and reports were reviewed at different levels in the organization to gather data on costs and outcomes of the programs. Patient card were reviewed to identify treatments direct costs of treatment for each beneficiary. The financial records and statements from Save the Children Ethiopia Country Office in Addis Ababa, Awassa EHNP office and Project Units were also reviewed.

### Method of cost estimation

The societal perspective analysis, which considers costs to all sectors of the society, was used. Collection of cost data included both the direct costs to the operations of the program and opportunity cost (economic cost) of the two alternative models under consideration for the cost-effectiveness analysis. The list of resources that were utilized in the programs in the period of the program were quantified or inventory reports secured from stock records and direct costs were estimated based on patient card review and recall of caretakers. Financial costs were also used to estimate actual economic costs in cases where direct economic costs were difficult to estimate. Since the cost estimation exercise in this study included use of cost data at different time periods, adjustments were made to account for inflation using appropriate indexes, GDP deflators and exchange rates [18,19].

For estimating costs of resources for which costs were not attached in the financial documents, current cost estimates were obtained. E.g. for drugs average costs were identified from international drug price guide 2005 and a document prepared for budget planning in the organization, based on the local prices from government and private suppliers in the area/Awassa in 2006. Similarly, for buildings local renting prices were used and for labor local payment rates reported from in-depth interviews and discussions.

### Measuring Effectiveness

For different alternatives included in CEA, a single effectiveness measure has been used. The effectiveness of the two programs thus was measured in terms of cure rates, or number of children cured from SAM as identified from their follow up care records. Other operational indicators of effectiveness such as rates of defaulters, non response, medical referral and mortality were provided. In this analysis a child was consider to be cured if a child is discharged fulfilling the criteria of Weight for Height ≥85% for two consecutive weighing and no edema for ten days.

### Cost inclusion and exclusion criteria

The cost of treating adults and moderately malnourished children cases (in SFP of the CTC) was excluded from the cost calculation in both programs as it is beyond the scope of this study. In areas where economic costs were found difficult to calculate, financial costs were used to approximate the estimation of opportunity costs especially for temporary shelters, equipments and constructions. Cost data that did not have any practical implication on program delivery such as cost of evaluation of programs, cost for community level water and sanitation schemes and others were not included in the study.

Cost of donations and volunteer work were estimated and included in the analysis though they may not be found in the financial records.

### Data Analysis and ethical issues

The quantitative data from the household survey and patient records were cleaned, entered into SPSS 12.0.1 and edited. The secondary data with regard to costs and effects of the programs were entered in to Microsoft excel spreadsheet, were cleaned, edited and analyzed. Uni-variate analysis was done and descriptions of data were given in tables for both costs of the programs. Computation and Comparison of cost effectiveness ratio was done using the average cost per child treated in the respective program.

Ethical clearance for the study was obtained from the ethical clearance committee of the Jimma University. Verbal informed consent was obtained from all participants.

## Results

### Socio demographic characteristics of study subjects

A total of 328 patient cards/records of children cured in the programs were reviewed; out of which 306 (157 CTC and 149 TFC) were traced back to their households to interview their caretakers. Many socio-demographic variables of the caretakers and households of the children under study did not have significant differences based on the model in which they were treated (TFC or CTC). Children in TFC were older (P < 0.001) and heavier (p < 0.001) at admission than children in CTC. Considering their location of residence, 46%, 53% and 58% of all the cases were near (4 hrs walking), medium distance (5-8 hrs walking) and far (> 8 hrs walking) taking Morocho TFC as a reference. The distribution of cases in this regard is more or less similar/homogeneous in both treatment groups (P value = 0.07). However, slightly more cases in the middle strata were treated in CTC than the TFC groups (P = 0.024). (See Table 1)

**Table 1:** Characteristics of 306 children affected whose treatment was costed, Shebedino Woreda, Sidama zone, March 2007

Characteristics	TFC (n = 149)	CTC (n = 157)	P-value
Mean age ± S.D. (months)	59.4 ± 47.8	41.42 ± 20.58	< 0.001
Mean admission weight ± SD	11.15 ± 8.02 Kg	8.77 ± 2.58 Kg	< 0.001
Sex (%males)	54.6	54.9	0.091
Sex of caretaker(%female)	58.1	76.1	< 0.001
Mud floor (%)	84.1	88.4	0.066
Mean length of stay in TFC/OTP ± SD	26.9 ± 10.9	42.7 ± 21.6	P < 0.001

### Institutional cost

#### Cost for routine and medicines for treatment of complication

The two programs were run by a humanitarian organization and therefore no user fees were incurred on the caretakers and families of the children. The routine medical cost was $2.13 per child for TFC and $1.76 per child for CTC.

Among the children treated in CTC, only 19 (11.6%) had to seek care in the Stabilization Center (SC). The average length of stay of a child admitted to SC was 13.3 days. The rate of infection in CTC was 12.8% (21 children). The cost for additional treatments for these medical problems/complications in the in CTC was estimated at $0.17 per child.

In the TFC group 71(43.3%) children were treated for complications. In comparison a significant number of children in TFC group had medical complications than CTC children (P < 0.001). The cost for additional treatments for these medical problems/complications in TFCs was estimated at $0.38 per child.

Generally, the average cost for medicines in CTC was $1.92 per child, less than in the TFC that is $2.51.

### Cost of therapeutic food

In this study the cost of therapeutic food provided per child was found to be $42.94 for TFC and $55.53 for CTC.

### Cost of Food for caretakers

As much of the supplies to provide food for caretakers came from organizations other than SC/USA the cost estimates from financial records could not reflect the real economic costs. Therefore, current costs were estimated based on a minimum meal budget for people in centers/campus/in the country which is $0.43 per day on average. The average cost of caretaker food was, thus calculated at $0.43 per day per caretaker. Thus, the total cost of food for caretakers in TFC was $11.64 per child. On the other hand, caretakers' food in CTC was $0.15 per child treated in CTC.

### Cost of Non food items provision

In the TFC, soap, a jerry can, a blanket, a pair of bed sheets and ITN given to the beneficiaries while in the center and at discharge that cost $23.25 per child. Some of the items were also given to CTC beneficiaries and a bucket to carry the bimonthly dry ration. The average unit cost of non-food items provided in this model (CTC) was $13.77.

### Personnel cost

In the TFC, health officers and nurses were responsible for the medical care of the children making physical examinations, ordering treatments and administering them. On the other hand in the a nurse from the Health Center paid regular visits for medical care of children in CTC for about 2 hrs every day. And the care of children in Outpatient sites was by nurses from the clinics and health posts and community volunteers. In addition administrative personnel at level were included in the cost estimation.

All in all average unit cost of staff cost per child for TFC ($122.36) was more than three times that of the CTC ($37.1).

### Capital depreciation and utilities

Based on the financial record reviews and reports for the period under study, capital depreciation and utilities costs of the programs were estimated. The space used for treatment, capital items, utility at the sites and SC, vehicle operation and supplies were included in this cost category. The medical equipments that are used in the programs were considered as recurrent expenditures assuming that with in the setting of emergency therapeutic feeding their life is not expected to exceed one year. Local renting prices were used as proxy for the current cost of buildings used in both programs. Similarly, the costs to run the program together with six other TFCs and CTC programs at Awassa and Addis Ababa offices were estimated and allocated to the specific programs.

The capital depreciation and utilities cost of the TFC was $50.47 per child and the CTC cost was $17.92 per child. A major portion of the overhead costs in both programs went to vehicle rental. Here also, TFC costs were about 3 times more than CTC costs.

### Total instructional costs

The total cost incurred on the intuition side to treat a child was calculated by adding all cost categories discussed above. The institutional cost in TFC was $262.62 per child. This shows that the average institutional cost of the TFC was more than twice that of the CTC program. (Table 2)

**Table 2:** Institutional cost in the two models, Shebedino Woreda, Sidama Zone, March 2007

Cost category	Therapeutic Feeding Center		Community-based therapeutic care	
	**Mean cost per child($)**	**Percent**	**Mean cost per child($)**	**Percent**
All personnel salaries	122.36	46.59	37.1	28.85
Capital depreciation and utilities*	50.47	19.22	17.92	13.94
Medicines	2.51	0.96	1.92	1.49
RUTF/Milk based formula	42.93	16.35	55.53	43.19
Caretakers' food	11.64	4.43	0.15	0.12
Non food item supplies	23.25	8.85	13.77	10.71
Other supplies	9.46	3.60	2.18	1.70

Total institutional cost	262.62	100.00	128.58	100.00

*Here utilities include vehicle fuel and operation, electricity, maintenance, etc, RUTF ready to use therapeutic food

### Costs to caretakers

#### Direct costs to caretakers

Caretakers and families spend money while seeking treatment for the child. These include costs of transportation, food and lodging. Generally, the average costs to caretakers for the TFC ($1.45 per child) were more than 2 times higher than the CTC ($0.92 per child).

### Opportunity cost to caretakers

The cost of lost productive time of caretakers while in program was calculated based on their occupational status and the total time spent on caring for the child during treatment. The monetary equivalents of these lost times were estimated using the rates currently paid in the localities for daily laborers from qualitative data. No difference was reported in the payment between sexes for similar jobs in the group discussions and in-depth interviews.

Assuming that those eligible caretakers to be productive all the time, the total lost productive earning of the caretakers, the opportunity in TFC was $20.92 per child and in CTC $5.88 per child. In the CTC group, caretakers' opportunity costs in terms of lost earning were substantially lower by about 3.5 times.

Combining the direct expenditure with the opportunity costs, the economic costs to caretakers, was about $21.93 per child for the TFC and $6.29 per child treated in CTC.

When the combined caretakers' and intuitional costs are considered, the TFC costs $284.56 per child and CTC $134.88. This shows that community based CTC was two times more cost effective than TFC. (Table 3)

**Table 3:** Cost of community-Based therapeutic care compared to inpatient Therapeutic feeding Centers

Cost category	TFC	OTP	Difference
	
	Mean cost per child($)	Mean cost per child($)	Mean cost per child
Total institutional cost	262.62	128.58	134.04

Direct caretakers cost	0.92	0.42	0.50

Caretakers opportunity cost	21.01	5.87	15.14

Total cost	284.56	134.88	149.68

### Outcomes of the interventions

A review on the monthly statistical and narrative reports of the two models revealed that both programs were up to or even exceeded the sphere project minimum standards in disaster response. There were a total of 646 children admitted to Morocho TFC. And of these 616 children were cured with cure rate of 95.36% there was no reported death in the TFC.

Out of the 649 children discharged from Shebedino CTC during the period under review, 612 were cured. The cure rate was 94.30% for CTC. There have been rather similarity as far as defaulters and non-response rates were concerned. The death rate in CTC (8, 1.2%) was higher than the TFC(0%). (Table 4)

**Table 4:** Treatment outcomes of Morocho TFC and Shebedino CTC, Sidama Zone, March 2007

Statistical indicator	TFC(n = 646)		CTC(n = 649)	
	**Number**	**Percent**	**Number**	**Percent**

Cured	616	95.36%	612	94.30%

Defaulted	8	1.24%	7	1.08%

Dead	0	0.00%	8	1.23%

Transferred to other TFC/CTC	16	2.48%	19	2.93%

Non responder	6	0.93%	3	0.46%

Total discharged	646	100.00%	649	100.00%

When cost per cured case is chosen as indicator, the average cost of TFC is 320 USD compared to 145.5 USD for the CTC. Therefore the average cost to cure a child is 2.5 times more in TFC than CTC, making CTC a more cost effective model of treating malnutrition in this area.

## Discussion

The comparison of the cost effectiveness of the two models should consider the difference in the context where they were implemented. Morocho TFC operated during a serious emergency and it was the first experience for the organization in such rapid response activities [21]. The CTC was implemented by the same organization after three years of operation in nutrition, and it was a second time intervention for the Woreda. Children in the TFC were significantly older and heavier than children in CTC at enrolment.

The findings in this study show that slight difference in socio-demographic status of caretakers in the two programs exists. The finding from this study revealed that there were 2.3 times more female caretakers (76%) in the CTC than in the TFC (58%). The reason behind this could be that mothers are busy with household chores and with the caring other children at home that are considered a priority in the community. In contrast, in the CTC model, which tends to demand only a fraction of women's time away from home in a day, female caretakers were more. This could also explain why, fathers of the children were taking care in TFC more frequently than mothers.

Caretakers direct costs were higher in the TFC than in the CTC group. Since TFCs are far from home, they tend to spend much money to reach the centers as compared to nearer to home distribution sites of CTC [30]. The average length of stay of children in the TFC (≈27 days) and CTC (≈42 days) was almost similar to the findings from other areas of the country and other countries [31].

From the direct cost of treatment supplies, cost of medicines used to treat complications was three times higher for TFC. This finding is in agreement with the findings from other studies, which indicate that TFCs pose risk of spread of communicable diseases [6,7]. The cost of therapeutic food to children was the only cost where the CTC model took the upper hand from all cost categories. The greater length of stay and lower rate of weight gain as compared to the TFC children might explain this [32]. Although the expected weight gain with such intake of RUTF, or any RUTF would be higher though sharing is a potential threat at home.

However, TFC food cost being lower is a different finding from reports of European Commission Humanitarian Aid Organization (ECHO) that compared costs per child per month. They estimated child food cost of 25€/child/month in CTC compared to 28€ in TFC. This included costs of SFP and was based on the budget proposal of agencies rather than reports [33]. However it can be seen from ECHO data, if we further break down this to cost per day, a similar trend with this study will be seen. In this study where the average length of stay is low in TFC, cost is low and as average days of stay is higher in CTC, cost is higher.

The significantly higher average cost of caretakers' food in the TFC might be due to a smaller proportion (11%) of children in CTC that required inpatient treatment. And even those admitted were discharged on average after 13.2 days when the child is treated of its medical problems and can take RUTF. This also applies to the fact that lower cost is incurred to material support of beneficiaries in CTC, as they are not mostly treated as in-patient.

Three times more cost was incurred for professionals in TFCs, as they are large centers of intensive care; the skill of professionals required is higher. Seventy-one full time support staffs were required to keep the center up to the standard working in three shifts round the clock. The staffs that ran the CTC however were only two staff, volunteers and part-time workers. This has an added advantage of building the capacity of the community and sustaining the effect of intervention after handing over to the local government. The significant difference in the personnel cost of TFCs was attributed to many expatriate staffs experienced in the programming of such activities were employed.

Moreover, coordination offices were established both at Addis Ababa and Awassa level compared to one coordination office at Awassa in the case of the CTC that require a significant number of administrative staff and running cost. When TFC overhead costs at Addis Ababa level were removed from the calculation, the difference in overheads between the two models was still maintained, but at lower degree (TFC was higher by two fold). More than 20% of this overhead cost was for international staff.

The share of cost to vehicle rental took a significant portion of the overhead cost in CTC, as logistic for the CTC were transported everyday to the sites with the team. In general, total institutional cost of treatment in TFC was substantially higher than in CTC by more than two times. Although all average costs in this cost categories except child's food were higher in absolute terms, child's food took the major portion of institutional costs in CTC.

Caretakers' costs (both direct and opportunity cost of lost productive time) were also 3.5 times higher in TFC. This might be because caretakers spent many days (about 27 days) out of home with their child in TFC. The cost to travel to the center was also remarkably higher than transport costs incurred by caretakers in CTC. The lower expenditure for drugs at other places in the CTC group may be because of the community mobilization and outreach activities helping families or community volunteers to detect the malnutrition situation and seek early treatment in the correct place/CTC.

In our study the cost of treating a child in CTC costs $134.88 and death rate of 1.2%. In addition CTC approach is more cost effective than the traditional-TFC; this is in agreement with many other studies [22,24]. According to recent review of costs of ambulatory community-based treatment of severe acute malnutrition have ranged between US$46 to $453 per child [34]. Recent studies have reported on costs and outcomes of similar large-scale African programs covering geographically defined populations, with ambulatory care for most children, and initial in-patient stabilization for the minority with most severe disease. In these studies the costs ranged from US$129 to $201 per child, and mortality rates ranged from 1.2 to 9.2%[34,35]. A decision tree model based on such a program in Zambia estimated that community-based treatment of severe acute malnutrition in primary-care centers, with hospital access, cost US$203 per case treated, compared with no treatment [36]. However it is lower than the ECHO report which said all things considered 329€/child is required for each grant. This may be due to the different nature of data used in this study [33].

This study is the first of its kind in this country. It has tried to employ a societal perspective of analysis. Caretaker side costs were included to enable their consideration in subsequent planning of the most cost effective intervention in the area. In this study caretaker side costs were estimated based on household survey, which increase validity of the estimates. Additionally, qualitative data were used to supplement the quantitative findings, which are important in any CEA [24]. Although the data were retrospective, all of them were adjusted to current value.

The main limitation of the study includes the study was mainly based on retrospective information, recall bias may appear especially in the estimation of opportunity cost to caretakers. Similarly, the record reviews were incomplete in some cases and accuracy was assumed in all cases once the relevant staff in the organization confirmed it. The other limitation was that the estimation of costs to caretakers in terms of productivity losses assumes similar pattern of work and did not take the seasonal variation of work availability in to consideration. Sensitivity analysis was done. Finally the baseline characteristics of the children in the two programs are different; children in CTC are younger and smaller at admission which might have implication on the program outcomes. In addition this limits the quantification of uncertainties.

## Conclusion

The findings suggest that community based therapeutic care is cost effective than inpatient therapeutic care. This also supports the view that CTC properly handled can save lives as much as TFCs. The major part of costs to treat a child in TFC went to administrative overhead and thus CTC was found to be more cost effective with many other positive impacts. Since therapeutic food costs were the significant part of the costs in CTC, local production of RUTF could cut the costs of care. Further comprehensive and prospective studies in drought prone pastoralist areas or socio-culturally different populations are recommended.

## Competing interests

The authors declare that they have no competing interests.

## Authors' contributions

AT, MW and GA designed the study and collected the data. AT and KD anlyzed the data and drafted the manuscript. All authors read and approved the final manuscript.
